# Supplementary material for: A Strategical Improvement in the Performance of CO2/N2 Gas Permeation via Conjugation of L-Tyrosine onto Chitosan Membrane
Source: Membranes (Basel). 2023 Apr 29;13(5):487. doi: 10.3390/membranes13050487 (PMC10223707; doi:10.3390/membranes13050487)
Supplement: Supplementary file 1 [file membranes-13-00487-s001.zip › membranes-2264822-supplementary.pdf]

Supporting Information

A Strategical Improvement in the Performance of CO_2/N_2 Gas Permeation via Conjugation of L-Tyrosine onto Chitosan Membrane

Aviti Katare [1], Rajashree Borgohain [1], Babul Prasad [2,*] and Bishnupada Mandal [1,*]

[1] Department of Chemical Engineering, Indian Institute of Technology Guwahati, Guwahati 781039, Assam, India
[2] William G. Lowrie Department of Chemical and Biomolecular Engineering, The Ohio State University, Columbus, OH 43210-1350, USA
* Correspondence: prasad.136@osu.edu (B.P.); bpmandal@iitg.ac.in (B.M.)

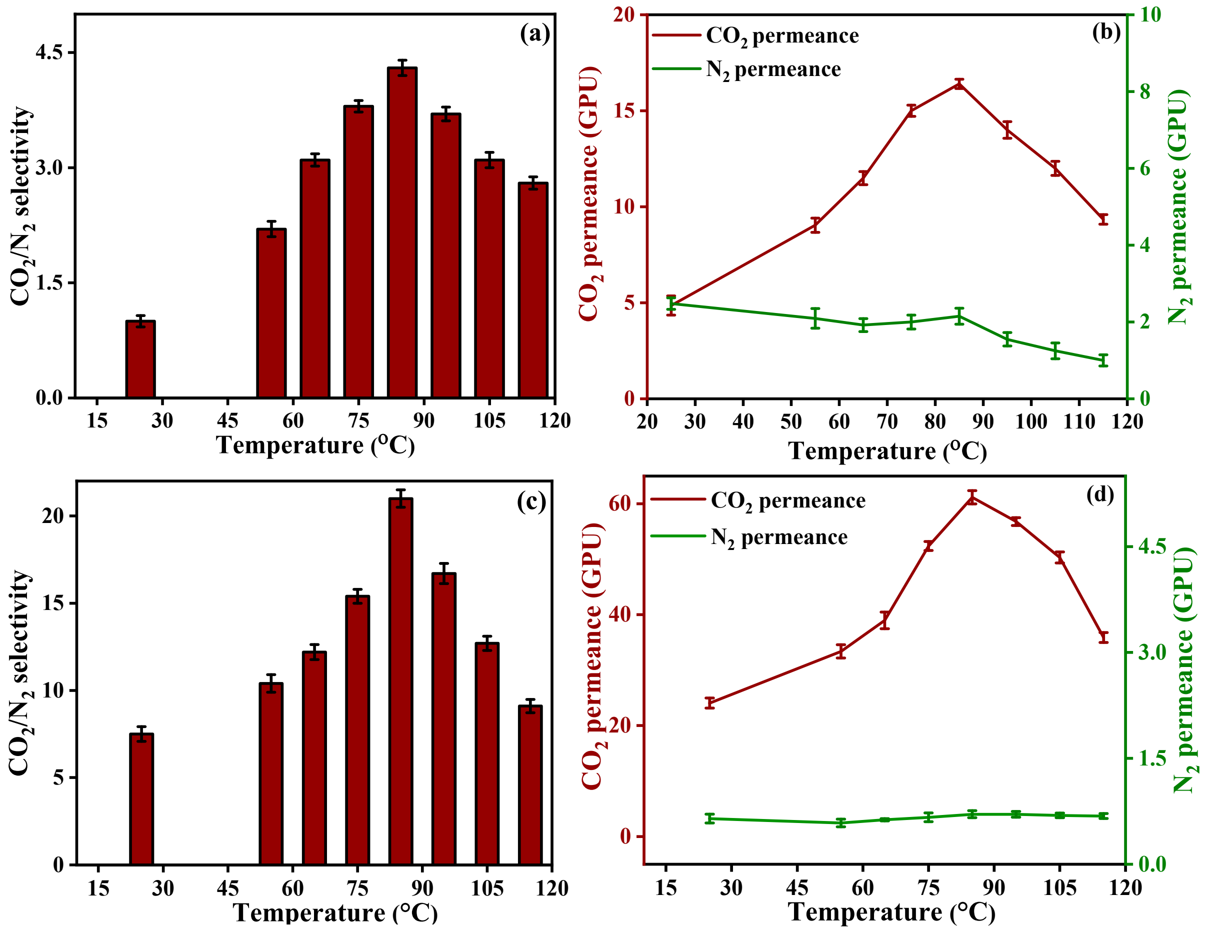

Figure S1. Effect temperature on (a) CO_2/N_2 selectivity, and (b) CO_2, N_2 permeance of dry CS membranes; Effect temperature on (c) CO_2/N_2 selectivity, and (d) CO_2, N_2 permeance of swollen CS membranes.

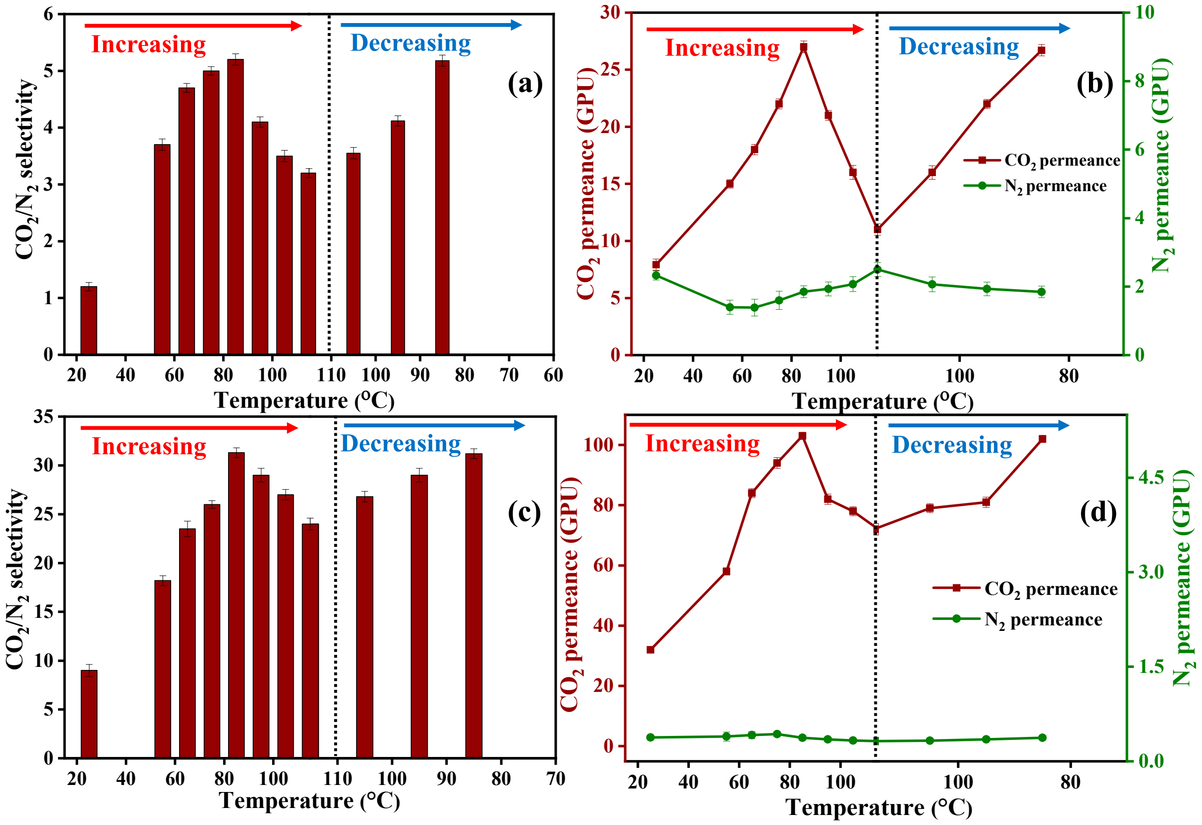

Figure S2: Effect of increasing and decreasing temperature on (a) CO_2/N_2 selectivity, and (b) CO_2/N_2 permeance of dry Tyr-c-CS membranes; and on (c) CO_2/N_2 selectivity, and (d) CO_2, N_2 permeance of swollen Tyr-c-CS membranes to confirm its stability and reusability towards gas separation applications.

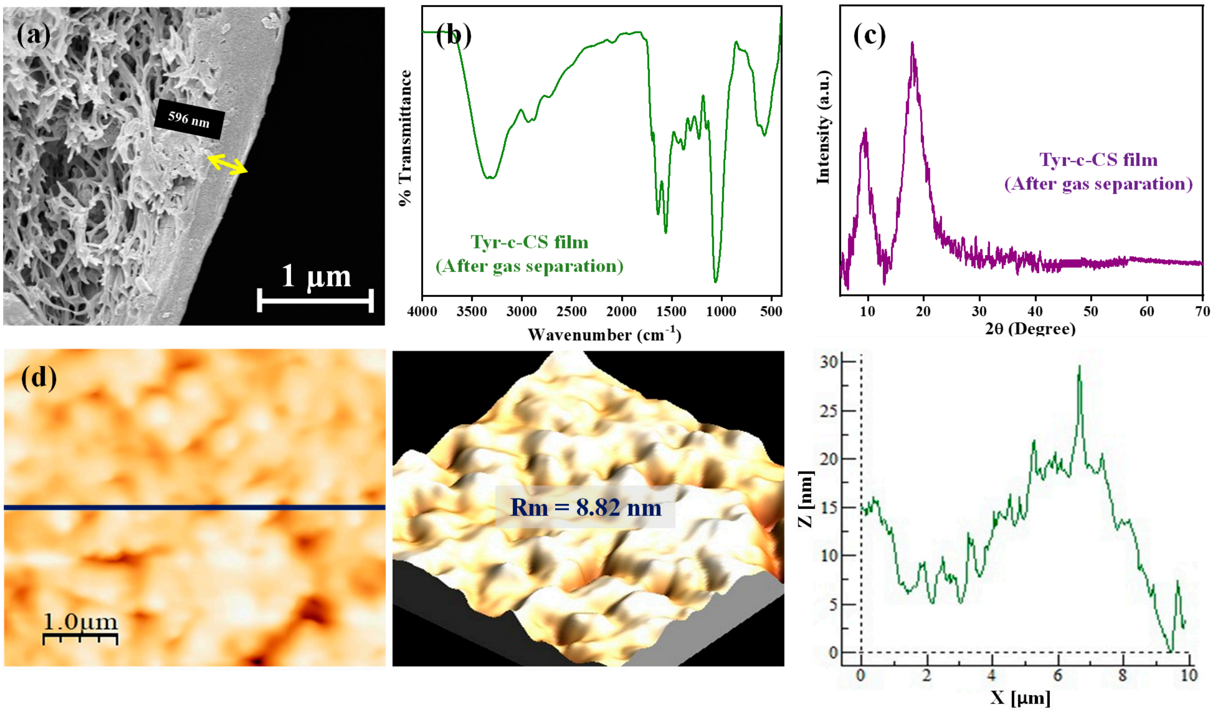

Figure S3: (a) FESEM image of cross section, (b) FTIR spectra, (c) XRD and (d) AFM image of top section of Tyr-c-CS membrane analysed after the gas separation experiments to confirm the stability and reusability of the membrane.
